# The effect of nebivolol and ramipril on selected biochemical parameters, arterial stiffness, and circadian profile of blood pressure in young men with primary hypertension: A 12-week prospective randomized, open-label study trial

**DOI:** 10.1097/MD.0000000000011717

**Published:** 2018-07-27

**Authors:** Marta Walczak-Gałęzewska, Monika Szulińska, Ewa Miller-Kasprzak, Danuta Pupek-Musialik, Paweł Bogdański

**Affiliations:** a Department of Internal Medicine, Metabolic Disorders and Hypertension; b Department of Education and Obesity Treatment and Metabolic Disorders; c Department of Physiology, Poznań University of Medical Sciences, Poznań, Poland.

**Keywords:** cardiovascular risk, hypotensive drugs, metabolic benefits

## Abstract

**Background:**

The pleiotropic effects of hypotensive drugs should always be taken into consideration. There is limited data on the effect of such drugs on reducing global cardiovascular risk in young hypertensives. The aim of this study was to evaluate the effect of nebivolol and ramipril on biochemical parameters, arterial stiffness, and circadian profile of blood pressure (BP) in young men undergoing treatment for hypertension (HT).

**Methods:**

A total of 80 patients aged 16 to 28 years of age with grade 1 HT were enrolled into the prospective randomized, open-label trial. They were randomized to receive 5 mg of nebivolol or 5 mg of ramipril, daily. Arterial stiffness index (SI), the circadian profile of BP registered in ambulatory blood pressure monitoring (ABPM), and biochemical parameters—including lipid profile, insulinemia, glycemia, and high sensitivity C-reactive protein (hsCRP) levels—were evaluated before and after the twelve-week period.

**Results:**

After the treatment period, we observed significant decreases in both ABPM systolic blood pressure (SBP) in group of nebivolol (*P* = .0007) and ramipril (*P* = .0001) and in ABPM diastolic blood pressure (DBP) in group of nebivolol (*P* = .0018) and ramipril (*P* = .0006). Reductions in the nondippers percentage were found in group of nebivolol and ramipril (*P* = .0077, *P* = .0001 respectively). Ramipril treatment resulted in a significant plausible modification in high-density lipoprotein (HDL) (*P* = .0390), glucose (*P* = .0213), and hsCRP (*P* = .0053) concentrations, as well as decreased SI (*P* = .0009) value, while nebivolol treatment showed no such benefits.

**Conclusions:**

Despite the similar hypotensive effect of nebivolol and ramipril, ramipril seems to possess better clinical potential in reducing cardiovascular risk in young men with HT.

## 1 Introduction

Hypertension (HT) is one of the most common diseases, occurring in over 20% of the adult population. HT is known to shorten life expectancy by about 5 years and is an important risk factor for other diseases, including heart failure, stroke, and renal diseases.^[1]^ The global epidemic of obesity has led to increasingly frequent HT diagnosis in younger patients. Based on data published in the literature, elevated blood pressure (BP) in adolescents is more common than previously thought. Cardiovascular prevention in young individuals should therefore be implemented. HT is a polyetiological disease, in which multiple coexisting disturbances are observed, including dyslipidemia, glucose impairment, and mild inflammatory state. Certain biochemical parameters—low-density lipoprotein (LDL), insulin resistance (IR), and high-sensitivity C-reactive protein (hsCRP)—are strongly associated with a higher risk of cardiovascular diseases.^[2,3]^ In the course of complex hypertensive treatment, HT-related disorders such as dyslipidemia and carbohydrates impairment should also be controlled. Younger patients are often unaware of HT, due to the lack of complications in early stages of the disease. The prevalence of HT in the Polish population between 18 and 39 years of age is 11% to 19% for men and 3.4% to 5% for women. The pathophysiological background of HT in younger adults differs from that found in older individuals. An important issue for adolescents is the higher plasma renin activity and increased adrenergic activity. Following the European and Polish Guidelines on Hypertension, there are 5 main groups of antihypertensive agents. We selected for this trial ramipril and nebivolol because they potentially have pleiotropic effects, can be safely administered (we did not include females in the study group because ramipril is known to have teratogenic effects), and have an acceptable trough–peak ratio. Given the scarcity of data on the effect of HT therapy using these drugs in young individuals, it was of great importance to perform an intervention aimed at preventing early complications in this group of patients.

### 1.1 Aim

The aim of this study was to assess the effect of nebivolol and ramipril therapy on selected biochemical parameters, the arterial stiffness index (SI), and the circadian profile of BP in young male hypertonics. To the best of our knowledge, this is the first study to evaluate these parameters in young men with HT.

Ethics approval and consent to participate was granted by the Research Ethics Committee of Poznań University of Medical Sciences (registered as no. 1027/09). Informed consent was obtained from all subjects.

## 2 Patients and methods

### 2.1 Study population

The study was designed as a prospective randomized, open-label trial, and was approved by the Research Ethics Committee of Poznań University of Medical Sciences (registration no. 1027/09). Informed consent was obtained from the subjects, and from the subjects’ parents if younger than 18 years.

Criteria for inclusion were as follows: age 16 to 28 years; male sex; newly diagnosed HT or HT that had not responded to previous nonpharmacological treatment; systolic blood pressure (SBP) in the 140 to 159-mm Hg range, or diastolic blood pressure (DBP) in the 90 to 99-mm Hg range based on office BP measurements (grade 1 HT). The exclusion criteria were as follows: documented secondary HT (confirmed by ultrasonography, angiocomputed tomography [angio-CT], assessment of hormonal factors inducing high BP, though not conducted in every patient), acute or chronic inflammation, heart failure, serious arrhythmias, diabetes, asthma, chronic obstructive pulmonary disease, chronic kidney disease (estimated glomerular filtration ratio [eGFR] <60 mL/min/1.73 m^2^), liver failure, electrolyte imbalance, mental retardation, statin intake; nicotine or alcohol abuse, or any other condition that, in the opinion of the investigators, would make participation not in the best interest of the patient or could prevent, limit, or confound the protocol-specified efficacy assessments.

Eighty young Caucasians aged 16 to 28 yaers with grade 1 HT were referred. They were screened at the Department of Internal Medicine, Metabolic Disorders, and Hypertension and Outpatient Clinic of Poznań University of Medical Sciences, and 60 individuals were found to meet all inclusion criteria and no exclusion criteria. These 60 participants enrolled into the study (with 30 placed into group of nebivolol and 30 into group ramipril), and a total of 57 men (28 receiving nebivolol and 29 receiving ramipril) completed the intervention; their data were analyzed for the study.

### 2.2 Planned intervention

The subjects were randomly assigned to group receiving 5 mg of nebivolol, or to group receiving 5 mg of ramipril. Randomization was performed by an independent statistician. The participants were randomly assigned (with a ratio 1:1) to receive 1 capsule of nebivolol (Actavis Group, HafnarfjörÐur, Iceland) or ramipril (Polpharma, Starogard Gdański, Poland) in the morning for 12 weeks. All drugs were packed in bottles without labeling.

Every 14 days, dietary intake was assessed on the basis of dietary intake interviews (24-hour recall). The intake of salt and caffeine consumption during the study was constant and comparable between groups. Physical activity was unchanged. The level of compliance required in terms of medical adherence and the above recommendations was 90%.

At the baseline, and after 12 weeks of treatment, the selected anthropometric and biochemical parameters, arterial SI, and the frequency of the dipper and nondipper HT patterns were determined for both groups.

### 2.3 Physiological, anthropometric, and biochemical measurements

Office BP was measured in line with the 2015 Polish Society of Hypertension Guidelines,^[4]^ using an Omron 705IT electronic device (Omron Healthcare Europe, the Netherlands). For 24-hour ambulatory blood pressure monitoring (ABPM), a BTL-08 ABPM device was used (BTL Industries, UK). Measurement frequencies of every 15 minutes during the day (from 6.00 to 22.00 hours) and every 30 minutes during the night (from 22.00 to 6.00 hours) were programmed. The overnight BP drop was calculated using the ratio: ([ABPM SBP] [day] − ABPM SBP [night]) × 100 /ABPM SBP (day). Patients whose nightly BP decrease was in the 10% to 20% range were assigned to the ‘dippers’ group, whereas those with a nightly BP decrease of less than 10% were assigned to the nondippers group. The results were interpreted only if at least 70% of the planned measurements were taken.^[5]^

Arterial SI was assessed using a PulseTrace PCA2 device (Micro Medical, Rochester, UK) using a photoplethysmographic transducer to measure the digital volume pulse.^[6,7]^

During anthropometric measurements, the patients wore lightweight clothing and no shoes. Weight and height were measured to the nearest 0.1 kg and 0.1 cm, respectively. Waist circumference (at the level of the iliac crest) and hip circumference (at the level of the greater trochanter) were measured. On the basis of these results, the body mass index (BMI) and waist-to-hip ratio (WHR) were calculated. Anthropometric measurements were taken at baseline due to the relatively short duration of the intervention.

Blood samples were taken in the morning after measuring SI and ABPM. Lipid profile, fasting glucose, and hsCRP were tested immediately using a Dimension biochemical system at the clinical laboratory in Poznań. The samples intended for insulin testing were frozen at −20°C and then evaluated using the immunoenzymating method (INS-EASIA, DIAsource ImmunoAssays, Louvain-la-Neuve, Belgium). Insulin resistance as Homeostasis Model Assessment-Insulin Resistance (HOMA-IR) was calculated with the formula: HOMA-IR = fasting insulin concentration [μIU/mL] × fasting glucose concentration [mmol/L]/22.5.^[8]^

### 2.4 Statistical analysis

The parameters were statistically described by their arithmetic mean, standard deviation (SD), and median. All parameters for analysis were tested for normal distribution using the Liliefors test. To compare values between groups, the unpaired *t* test was used or else the Mann–Whitney test applied (the variable is not compatible with the normal distribution). To evaluate changes in parameters within a group, the *t* test was used for dependent samples, or else, the Wilcoxon matched-pairs test employed (the variable is not compatible with the normal distribution). To examine the relationship between variables measured at nominal scale, chi-square or chi-square with the Yates correction test was used; the nominal McNemar test was used to examine the relationship between changes in the time variable. The sample size of 80 representatives was based on a confidence level of 95% with a margin of error 11%. The power calculation for unpaired samples was in the range 6% to 12%, and for paired samples was in the range 80% to 96%. The statistical analysis was performed using Statistica 10 (StatSoft, Tulsa, OK). The level of statistical significance was set at *P* <.05.

## 3 Results

Young male patients with grade 1 HT were randomized into 2 groups, receiving nebivolol (n = 30) and ramipril (n = 30), respectively. At baseline, no statistically significant differences in age or anthropometric parameters were observed between analyzed groups; they were comparable in most of the analyzed parameters, including lipid profile, insulinemia, glycemia, SI, and BP. There were no differences in the BP circadian profile. The concentration of hsCRP was higher in patients from B group allocated to the ramipril treatment (Table 1). Four participants from group A and 5 from group B fulfilled the criterion for obesity (BMI ≥30 kg/m^2^).

**Table 1:**
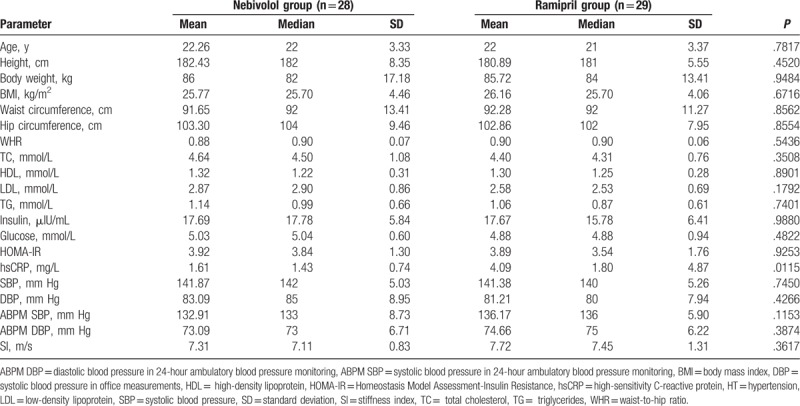
Baseline anthropometric characteristics, biochemical parameters, blood pressure in office measurements (SBP, DBP), 24-hour ambulatory blood pressure monitoring (ABPM SBP, ABPM DBP), and arterial stiffness index (SI) of the HT patients allocated to nebivolol or ramipril treatment.

**Figure 1:**
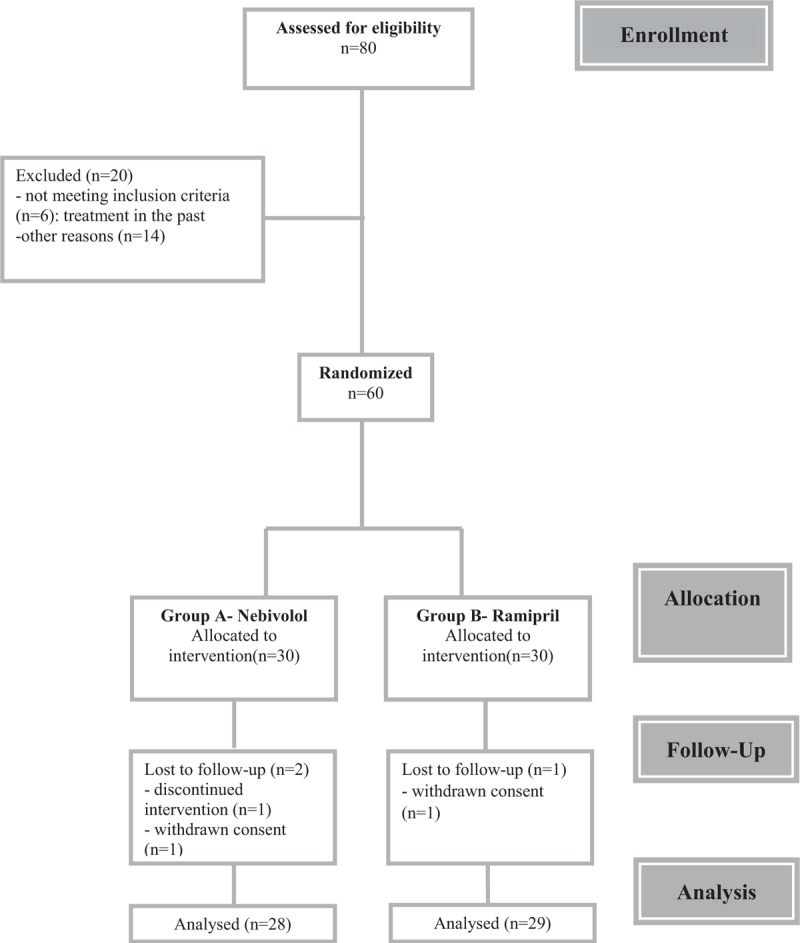
Consort flow diagram of the study.

After 12 weeks, group of patient administrating nebivolol contained 28 patients (1 had discontinued the intervention and 1 had withdrawn consent), whereas group of patient administrating ramipril consisted of 29 patients (1 had withdrawn consent). After the intervention, the data from the remaining 28 patients treated with nebivolol and 29 patients treated with ramipril were subjected to analysis (Fig. 1). No statistically significant differences with respect to biochemical parameters and SI before and after the 12 weeks of nebivolol treatment were found (Table 2). On the contrary, the ramipril intervention was associated with a statistically significant increase in high-density lipoprotein (HDL) concentration (*P* = .0390), and lowered glucose (*P* = .0213) and hsCRP concentration (*P* = .0053). Trends toward a decrease in the concentration of insulin and HOMA-IR were visible, but did not obtain statistical significance (Table 3). A significant reduction in the SI value (*P* = .0009) was observed after ramipril intervention.

**Table 2:**
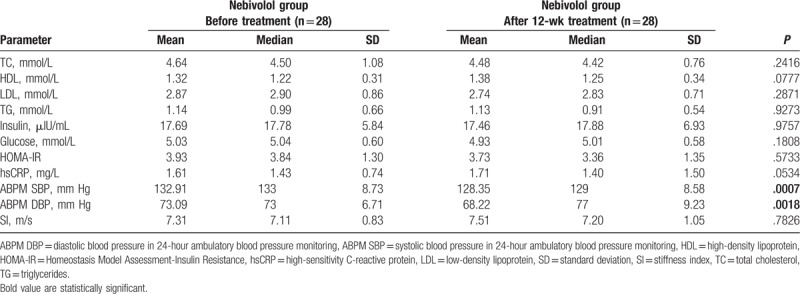
Biochemical, blood pressure in 24-hour ambulatory blood pressure monitoring (ABPM SBP, ABPM DBP), and stiffness index (SI) characteristics of the nebivolol group before/after 12-week treatment.

**Table 3:**
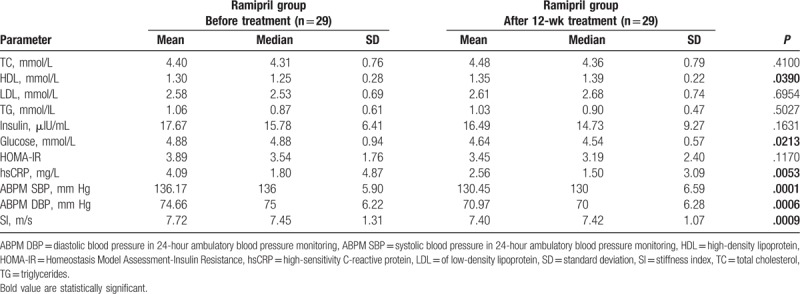
Biochemical, blood pressure in 24-hour ambulatory blood pressure monitoring (ABPM SBP, ABPM DBP), and stiffness index (SI) characteristics of the ramipril group before/after 12-week treatment.

After the treatment period, we found significant decreases in ABPM SBP for group of nebivolol (*P* = .0007) and group of ramipril (*P* = .0001), and decreases in ABPM DBP for nebivolol group (*P* = .0018) and ramipril gropu (*P* = .0006) (Tables 2 and 3).

In both groups, the dipper/nondipper HT status changed after 12 weeks of treatment, and there was a statistically significant increase in the percentage of dippers (nebivolol group: *P* = .0077; ramipril group: *P* = .0001; Table 4).

**Table 4:**
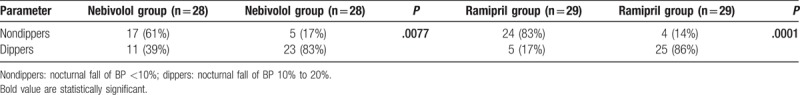
Dipper/nondipper status of HT after intervention with nebivolol and ramipril.

A comparison of the delta values of the variables (before and after treatment) revealed significant differences in the mean delta values of the heart rate (HR) (*P* = .0001) and SI (*P* = .0218) in the nebivolol group, as compared with the variables in the ramipril group (Table 5).

**Table 5:**
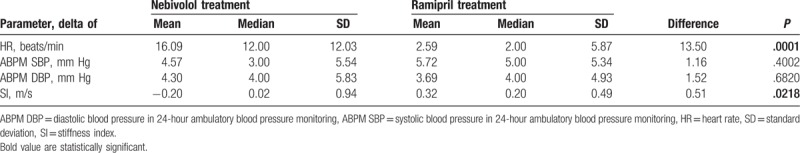
Comparison of the delta values of the variables (before/after treatment).

## 4 Discussion

We observed in our study that ramipril presented a better pleiotropic profile than nebivolol. Nebivolol and ramipril, as expected, had similar hypotensive effects and. After 12 weeks of treatment, for both drugs, we saw a statistically significant reduction in the percentage of nondippers, which can be counted as a positive effect of the treatment that is likely to reduce left ventricular hypertrophy, inhibit the progression of albuminuria and of atherosclerosis, and reduce the risk of cardiovascular events.^[9,10]^ Pickering estimated that lack of nocturnal lowering of BP occurred in approximately 20% to 30% of patients with HT.^[11]^ Cuspidi et al^[12]^, in a group of 229 hypertensive individuals, counted 51.9% to be nondippers. However, it should be emphasized that, although the ABPM measurements have higher reproducibility than the traditional BP assessment, they are not completely accurate, which may result in a change in the proportions of HT status in particular studies.^[9]^

In contrast to many studies that have pointed to the favorable metabolic profile of nebivolol,^[13–17]^ our study showed no statistically significant changes in lipid profile, insulin, glucose, and HOMA-IR after nebivolol treatment. This discrepancy may be due to several factors, including the small size of our group, the duration of the intervention, and the different profile of hypertensives (young age, lack of metabolic syndrome). Conversely, in the ramipril-treated group, we observed beneficial changes in biochemical parameters including HDL, glucose, and hsCRP concentrations. There are numerous data indicating the positive effect of ramipril on the lipid profile. In line with our results, Kyvelou et al^[18]^ showed a significant increase in HDL concentration, and a similar observation was reported by Salardi et al.^[19]^ During the follow-up at week 12, we also confirmed that there was a beneficial effect of ramipril treatment on carbohydrate metabolism in the form of a significant reduction in glucose levels. We also observed a significant reduction in hsCRP concentrations. Verma et al compared the effect of 10 mg of ramipril on CRP levels in 132 healthy volunteers (with baseline CRP ≥2 mg/L) in a placebo-controlled study. After 12 weeks of ramipril administration, they noted significant reductions in the average concentration of CRP, from 3.68 to 2.89 mg/L.^[20]^

One of the important parameters that identify subclinical organ damage is SI. Safar^[21,22]^ demonstrated higher values of SI in hypertensives. Millasseau et al^[23]^ showed that SI correlated with carotid-femoral pulse wave velocity (cfPWV), a gold standard of arterial stiffness. A clinically important finding of our study is the dissimilar effect of particular antihypertensive drugs on arterial wall properties and changes in SI. It has been observed that nebivolol improves artery stiffness through its vasodilating properties and its favorable effects on lipid and carbohydrate metabolism, AND ALSO on endothelial function and oxidative stress.^[24]^ Mahmud and FeEly showed that treatment with nebivolol reduced arterial stiffness in 50-year-olds. In contrast, our 16 to 28-year-old patients treated with nebivolol saw no reduction in SI values, suggesting a possible effect of age on the results. Nebivolol is a racemic mixture of the d-enantiomer, responsible for β-blocking, and the l-enantiomer for vasodilation.^[25,26]^ Taking into the consideration the short period HT has lasted in these patients, the slight changes in the arterial wall, and the increased adrenergic activity, the hypotensive effect of nebivolol in our subjects might be caused by β-blocking. In the group treated with ramipril, we noticed a statistically significant reduction in SI values. Similar findings were reported by Ahimastos et al in a study comparing the effects of ramipril versus placebo in a group of 44 patients with peripheral arterial disease; after 6 months of treatment, they found a decrease in PWV in the ramipril group, as opposed to the placebo group.^[27]^

In our study, a comparison of the delta values reveals a significant difference in the mean delta SI between the groups treated with nebivolol and ramipril. We also found a statistically significant difference in the mean delta HR between the nebivolol and ramipril groups.

Hypertension is 1 of the most common diseases, although the younger population is very often not aware of it. It is very important to help them understand the nature and possible course of HT. The conviction that, apart from normalizing BP, we can also affect the biochemical parameters and inhibit subclinical complications, might be crucial in the course of HT pharmacotherapy in young individuals.

The limitations of our study are related to the small number of patients and the relatively short research period. The sample size, with its 11% margin of error, could also be considered a limitation, given its insufficient power to evaluate the therapeutic effects of either nebivolol or ramipril. We analyzed selected biochemical parameters, with no parameters describing renal function such as creatinine, uric acid, and eGFR, which is a relevant study limitation. The higher baseline hsCRP values in the group randomized to ramipril might have been caused by more frequent mild inflammation among these subjects, which is also a limitation of that study. We realize that such an important issue requires prospective studies with long-term observation.

## 5 Conclusions

Despite these limitations, we have shown that changes in artery walls can be stopped or even reduced, and the circadian profile of BP can perhaps be corrected in the course of tailored hypotensive treatment. In our opinion, changes observed in metabolic and inflammatory parameters, which are beyond statistical significance, are highly desirable. We have documented the fact that the effects of ramipril therapy on biochemical parameters may be more beneficial than nebivolol in young hypertensive adults.

## Acknowledgment

We would like to thank Dr Izabela Miechowicz for her statistical consultation.

## Author contributions

MWG, MS, and DPM designed the trial; MWG and EMK collected the data; MWG, MS, and EMK wrote the first draft of the article; DPM and PB revised it critically for important intellectual content; all authors participated in the final revision of paper; all authors have read and have given final approval of the version to be published.

**Conceptualization:** Danuta Pupek-Musialik and Marta WAlczak-Gałęzewska.

**Data curation:** Marta Walczak-Galezewska.

**Formal analysis:** Marta Walczak-Galezewska, Monika Szulińska, Ewa Miller-Kasprzak, Pawel Bogdanski.

**Funding acquisition:** Danuta Pupek-Musialik, Paweł Bogdański.

**Investigation:** Marta Walczak-Galezewska.

**Methodology:** Monika Szulińska, Ewa Miller-Kasprzak, Danuta Pupek-Musialik, Pawel Bogdanski.

**Project administration:** Marta Walczak-Galezewska.

**Software:** Ewa Miller-Kasprzak.

**Supervision:** Monika Szulińska, Danuta Pupek-Musialik, Pawel Bogdanski.

**Writing – original draft:** Marta Walczak-Galezewska.

**Writing – review & editing:** Marta Walczak-Galezewska, Monika Szulińska, Ewa Miller-Kasprzak, Danuta Pupek-Musialik, Pawel Bogdanski.
